# Preference-based versus randomized controlled trial in prostate cancer survivors: Comparison of recruitment, adherence, attrition, and clinical outcomes

**DOI:** 10.3389/fonc.2022.1033229

**Published:** 2022-12-12

**Authors:** Shabbir M. H. Alibhai, Efthymios Papadopoulos, Sara Durbano, George Tomlinson, Daniel Santa Mina, Paul Ritvo, Catherine M. Sabiston, Andrew G. Matthew, James Chiarotto, Souraya Sidani, S. Nicole Culos-Reed

**Affiliations:** ^1^ Department of Medicine, University Health Network, Toronto, ON, Canada; ^2^ Institute of Health Policy, Management and Evaluation, University of Toronto, Toronto, ON, Canada; ^3^ Faculty of Kinesiology and Physical Education, University of Toronto, Toronto, ON, Canada; ^4^ Department of Psychology, School of Kinesiology and Health Science, York University, Toronto, ON, Canada; ^5^ Department of Surgical Oncology, Princess Margaret Cancer Centre, University Health Network, Toronto, ON, Canada; ^6^ Department of Medicine, Division of Hematology/Oncology, Scarborough Health Network, Scarborough, ON, Canada; ^7^ Daphne Cockwell School of Nursing, Toronto Metropolitan University, Toronto, ON, Canada; ^8^ Faculty of Kinesiology, University of Calgary, Calgary, AB, Canada; ^9^ Department of Oncology, Cumming School of Medicine, University of Calgary, Calgary, AB, Canada

**Keywords:** prostate cancer, androgen deprivation, group exercise, home-based exercise, fatigue, functional endurance, preference trial

## Abstract

**Introduction:**

Patients’ unwillingness to be randomized to a mode of exercise may partly explain their poor recruitment, adherence, and attrition in randomized controlled trials (RCTs) of exercise in oncology. It is unknown whether a preference-based trial can improve recruitment, adherence, retention, and clinical outcomes compared to a RCT of the same exercise interventions.

**Objective:**

We assessed the effects of a 2-arm exercise preference trial on adherence and clinical outcomes compared to a similar 2-arm RCT in men with prostate cancer (PC).

**Methods:**

This was a two-arm preference-based trial of group-based training (GROUP) or home-based training (HOME). PC survivors on androgen deprivation therapy (ADT) who declined randomization to the RCT but chose to participate in a preference trial were recruited in four Canadian centers. All study participants engaged in aerobic and resistance training, 4-5 days weekly for 6 months, aiming for 150 minutes/week of moderate-to-vigorous physical activity. The primary outcomes were changes from baseline to 6 months in fatigue and functional endurance. Secondary outcomes were quality of life, physical fitness, body composition, blood markers, and adherence. Linear mixed models were used to assess the effects of HOME versus GROUP on primary outcomes. In pooled preference and RCT data, the selection effect (i.e., difference between those who were and were not willing to be randomized) and treatment effect (i.e., difference between GROUP and HOME) were estimated using linear regression.

**Results and conclusion:**

Fifty-four participants (mean [SD] age, 70.2 [8.6] years) were enrolled (GROUP *n*=17; HOME *n*=37). Comparable effects on primary and secondary outcomes were observed following GROUP or HOME in the preference-based trial. Adherence was similar between preference and RCT participants. However, attrition was higher in the RCT (50.0% vs. 27.8%, p= 0.04). Compared to GROUP, HOME was more effective in ameliorating fatigue (mean difference: +5.2, 95%CI=1.3 to 9.3 *p*=0.01) in pooled preference and RCT data. A preference-based trial results in comparable observed effects on clinical outcomes and adherence and lower attrition compared with a RCT of the same exercise interventions in PC survivors on ADT. Given the appeals of preference-based trials to study participants, additional studies are warranted.

**Clinical trial registration:**

clinicaltrials.gov, identifier (NCT03335631).

## Introduction

Randomized controlled trials (RCTs) provide the highest level of evidence for clinical interventions in the behavioral sciences, including exercise-based interventions. However, the validity of RCTs is contingent, in part, upon acceptable recruitment, intervention adherence, and retention rates. It is widely recognized that poor recruitment is a major challenge in RCTs (1–3) and may lead to delays in study completion, increases in costs, reduced study power, premature completion, and difficulties with publication (1). In exercise oncology research, recruitment rates in RCTs range from 25.4% - 48.7% (4–11) of eligible patients (4–6, 8, 9, 11). Compared to pharmaceutical RCTs, exercise trials often require substantial time commitments from participants, such as frequent travel to study centers for supervised intervention delivery and completion of study assessments. Common barriers to participation in exercise RCTs include lack of time, transportation, or interest, and unwillingness to be randomized (5, 11, 12). RCTs may thus consist of highly selected individuals who may be indifferent with regards to some exercise preferences and settings and may exhibit differences in socioeconomic status from the general population (e.g., more highly educated, of White race, and of higher income status) or underserved groups of patients, thereby reducing the generalizability and relevance of findings to broader clinical populations. Moreover, adherence is another essential component for reliably assessing the effects of the intervention(s) in a RCT. Evidence from a systematic review found moderate-to-high adherence in patients with advanced cancer (range in RCTs only: 67-93.2%) (12). However, it is currently unclear whether selection of the treatment arm in an exercise RCT can foster adherence in patients with cancer. In addition to recruitment and adherence, attrition may be a challenge that can negatively impact the investigators’ ability to draw conclusions based on an adequately powered RCT while impeding participants from deriving maximum health benefits from the intervention. Attrition has been shown to vary among exercise RCTs in oncology (range: 6-36%) (12, 13). Strategies to minimize attrition, including allowing participants to select their preferred exercise intervention, have had limited study to date.

The potential of participants’ preferences to optimize intervention acceptance and adherence in oncology may have a direct impact on the effects and magnitude of exercise benefits. In addition to adherence, other mechanisms that can positively influence study outcomes in preference trials may include higher motivation and positive affect, as well as other mechanisms that require further exploration. Contrary to this hypothesis, previous studies in mixed clinical populations have revealed no differences in study outcomes between randomized and preference groups (14, 15). Nonetheless, whether participants’ preferences can augment exercise benefits in oncology, to the best of our knowledge, is currently unknown.

The concept of preference-based trials is gaining increasing attention (16, 17), as it may overcome some barriers to participant enrollment in RCTs such as the setting of the intervention or an unwillingness to be randomized (14); it may also be a promising strategy for reducing health disparities among patient groups. Although preference-based trials may mitigate challenges with recruitment and potentially adherence (18), they may compromise the internal and/or external validity of a trial which can subsequently impact the interpretation of study findings. For example, threats to external validity may include the decision of participants to enroll in the study in addition to potential differences in baseline characteristics that do not represent the majority of the population (19). Threats to internal validity influenced by preferences include attrition, intervention adherence, and changes in study outcomes (19). Currently, the impact of preference exercise trials on adherence, attrition, and clinically relevant outcomes is poorly understood, particularly in patients with cancer. Given the low recruitment rates reported in exercise trials in oncology and the important role of participant preferences in optimizing recruitment and adherence, we conducted a preference trial in men on androgen deprivation therapy (ADT) for prostate cancer (PC) who were eligible to participate in a RCT of exercise interventions delivered in two different settings but declined to be randomized.

The study objectives were to: i) identify the proportion of patients who declined randomization but were willing to be enrolled in a preference trial; ii) compare baseline characteristics of patients who agreed to be randomized and those who declined randomization but selected a preferred intervention arm; iii) compare the effects on fatigue and functional endurance (co-primary outcomes), as well as on secondary outcomes, such as quality of life (QOL), physical fitness and blood outcomes in those with an expressed preference for a particular exercise; iv) compare adherence and attrition rates between participants in the preference trial and RCT; and v) estimate the selection effect (i.e., the effect on study outcomes of being in the preference trial or RCT, regardless of the mode of exercise delivery) and treatment effect (i.e., effect of each mode of exercise delivery, regardless of randomization) for the primary and secondary outcomes of the study. We hypothesized that baseline characteristics such as distance to study center, age, education, prior experience with participating in exercise programs, and fatigue will differ between participants who selected their arm of preference versus those who were randomized. Additionally, we hypothesized that adherence and benefits in QOL and physical fitness outcomes will be greater among participants in the preference trial compared with their RCT counterparts.

## Methods

**Figure 1:**
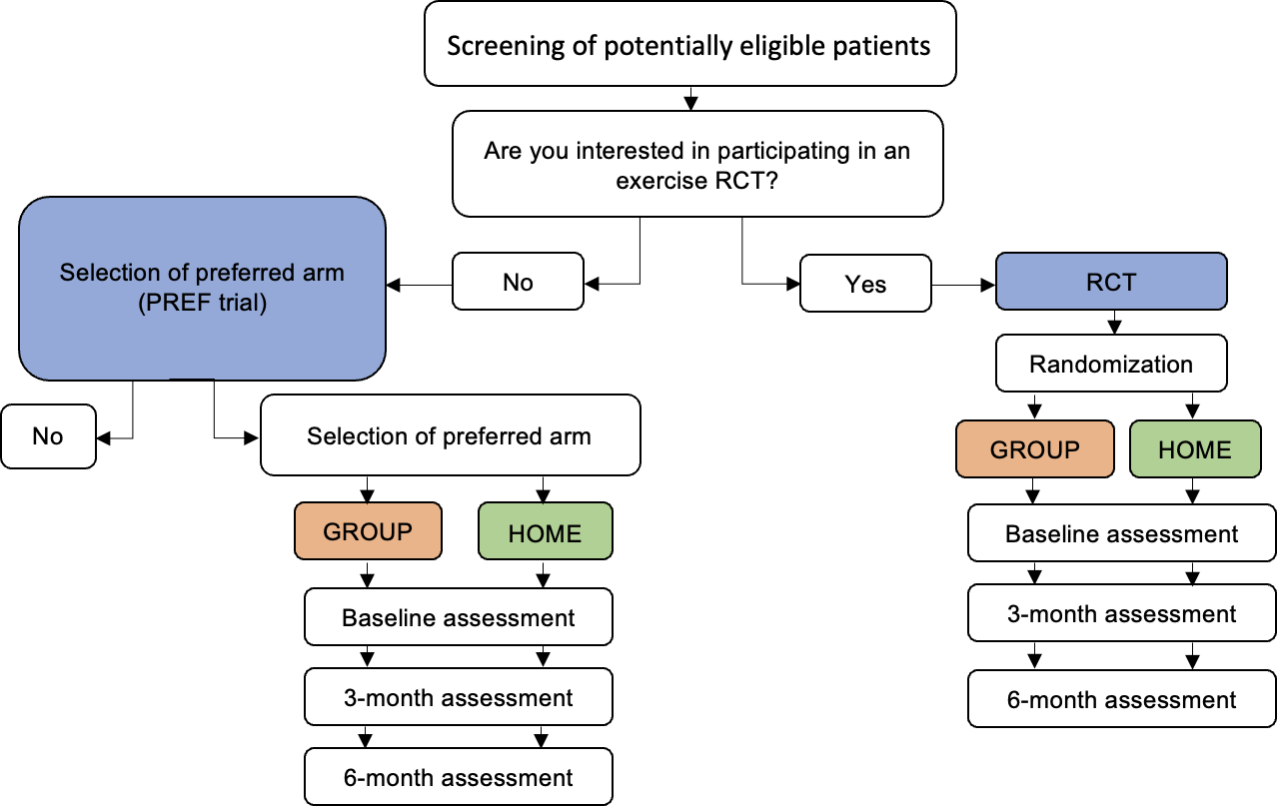
Schema of preference-based trial and RCT. RCT, randomized controlled trial; PREF, preference. Blue denotes comparison of the selection effect (PREF vs. RCT). Orange and green denote the comparison of the treatment effect (GROUP-PREF + GROUP-RCT vs. HOME-PREF + HOME-RCT).

This study was a multi-center 2-arm preference trial of in-person, group-based training (GROUP) versus independent home-based training (HOME) which was a companion to an open multi-center 2-arm non-inferiority exercise RCT (see **Supplemental Figure 1**) that involved the same interventions (i.e, GROUP vs. HOME) with blinded, validated, and clinically relevant outcome measures (**Figure 1**) (20). The RCT protocol has been published (20) and its results will be reported in detail elsewhere (manuscript in preparation). Participants for both trials [i.e., preference (from October 2017 until April 2020) and RCT (from August 2016 to March 2020] were recruited from two Canadian tertiary cancer centers and two Canadian regional hospitals. All study procedures were approved by the Research Ethics Board of the participating institutions and all participants provided written informed consent. This study was registered at clinicaltrials.gov (Registration #NCT03335631). To address our objectives, we describe the preference trial and its analysis in detail, and compare specific outcomes with the RCT.

### Participants

**Figure 2:**
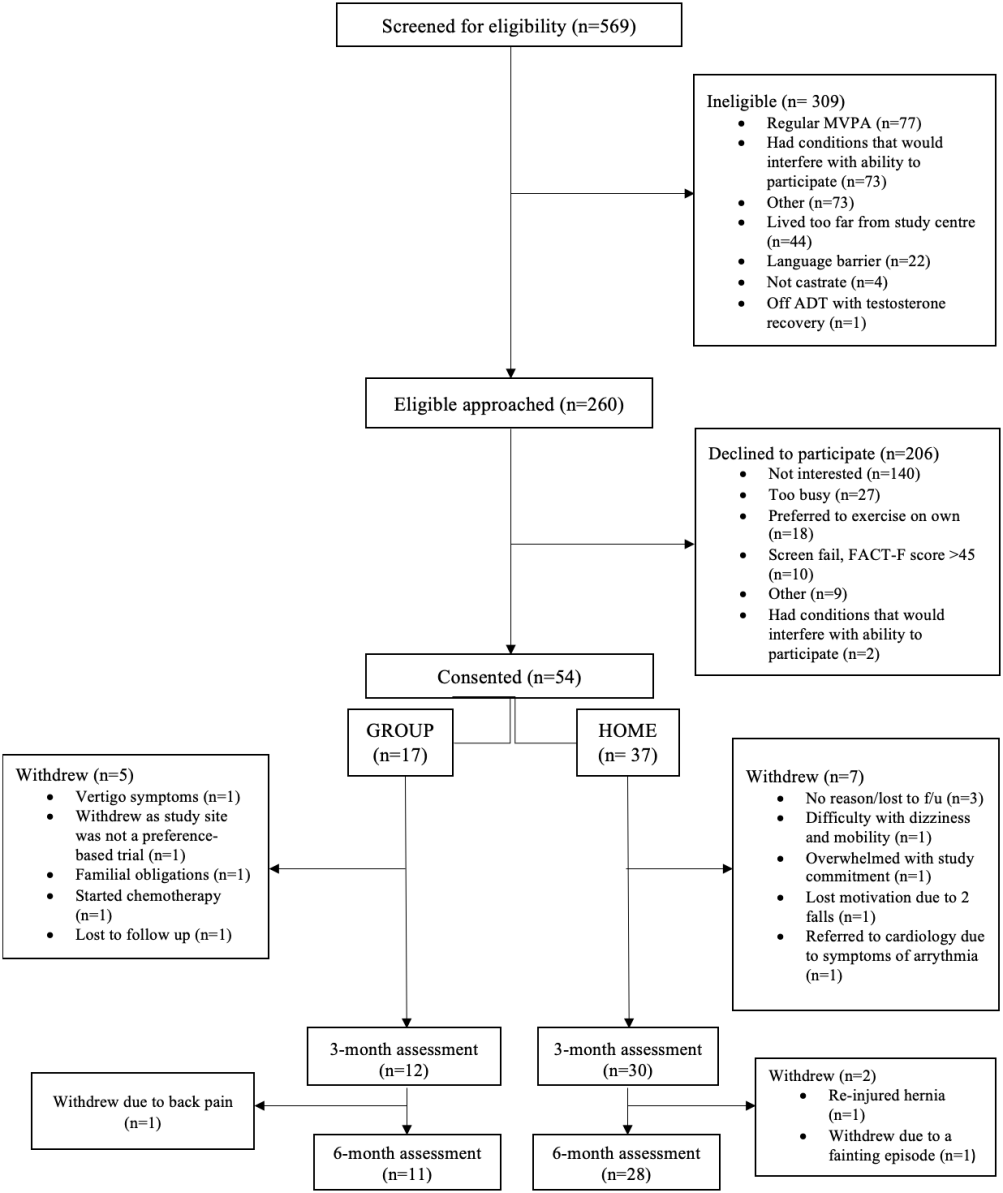
Flow diagram of study participants.

Study participants were PC survivors who were starting or continuing ADT for at least 6 months and were able to communicate in English. Exclusion criteria involved meeting physical activity guidelines and specific medical conditions that would potentially interfere with study participation. Details on inclusion and exclusion criteria are published elsewhere (20). All participants in this study were eligible but declined to be randomized to the phase III RCT (20) and reasons for declining participation were recorded by the research coordinator (**Figure 2**). Patients who declined to be randomized due to distance to the study center, an expressed preference for one of the two exercise delivery modes, or unspecified reason were offered participation in the exercise delivery model of their preference (the ‘preference trial’) and were asked to notify the research team about their decision. Patients could take as much time as they wanted to make a decision. When the preference trial opened, eligible men who previously declined the RCT were recontacted to see if they were interested in the preference trial.

### Intervention

Exercise delivery models included GROUP or HOME. Both exercise delivery modes comprised aerobic and resistance training 4-5 days per week for 6 months. After 6 months, supervised sessions and contact in both arms were tapered to facilitate independent exercise. The aerobic training component involved 30 minutes of participant-preferred aerobic modalities at 60-70% heart rate reserve. Resistance training consisted of upper and lower exercises of major muscle groups, using resistance bands, free weights, body weight, a stability ball and exercise mat. Participants were instructed to complete 2-3 sets of 8-12 repetitions at 60-75% of estimated one-repetition maximum. Each session concluded with 5-10 minutes of static stretching. The intervention arms including exercise parameters and duration in the preference trial were the same as those in the exercise RCT.

### Group-based training

GROUP participants had 3 exercise classes per week in groups of 4-8, supervised by a qualified exercise professional (QEP). Additionally, GROUP participants were provided with complimentary exercise equipment as described above and were asked to exercise independently 1-2 days weekly in addition to their group classes. To support participants’ transition to independent exercise, supervised sessions were reduced from 3 to 2x/week in months 5 and 6. GROUP sessions in the preference trial included only participants who selected GROUP while participants who were randomized to GROUP (RCT) engaged in separate GROUP sessions.

### Home-based training

Participants who selected HOME were asked to complete unsupervised, at-home exercise sessions 4-5 times per week using complimentary equipment that was provided by the study team. To facilitate exercise adherence and maintenance, HOME participants were in weekly contact with a health coach by phone or email and had one in-person session each month. Health coaches were QEPs or graduate students with training in health coaching. Communication between participants and health coaches addressed program-related barriers and informed progression of the exercise program. To aid communication and to track exercise-related metrics, we used the NexJ Connected Wellness (NexJ Systems, Inc., Toronto, Canada) application. Further, HOME participants also had access to complimentary informational video supports, smartphones, and Fitbits.

### Outcome assessments

Study outcomes were assessed at baseline and every 3 months for 12 months by non-blinded assessors. Months 1-6 represent the active intervention period, whereas months 7-12 correspond to the follow-up period, where sessions were tapered. Herein, we focus on intervention efficacy, which was assessed at 6 months. All study outcomes are described below.

### Primary outcomes

Study outcomes are listed in **Table 1**. Similar to the phase III RCT (20), co-primary outcomes included fatigue and functional endurance as measured by the Functional Assessment of Cancer Therapy-Fatigue (FACT-F) (21) and the 6-minute walk test (6MWT) (22), respectively.

**Table 1:** Study outcomes and time points.

Outcome	Baseline	3 months	6 months
***Co-primary***			
Fatigue (FACT-F)	•	•	•
Functional endurance (6MWT)	•	•	•
***Secondary***			
QOL (FACT-G & FACT-P subscale)	•	•	•
Upper body strength (grip strength)	•	•	•
Lower body function (5 chair stands)	•	•	•
BMI	•	•	•
Body composition (BIA)	•		•
Waist circumference	•		•
WC:hip circumference ratio	•		•
Fasting blood glucose	•		•
Total cholesterol	•		•
LDL	•		•
HDL	•		•
Triglycerides	•		•
PSA	•		•
Hemoglobin	•		•
HbA1c	•		•

6MWT, 6-minute walk test; BIA, bioelectrical impedance analysis; BMI, body mass index; FACT-F, Functional Assessment of Cancer Therapy – Fatigue; FACT-G, Functional Assessment of Cancer Therapy – General; FACT-P subscale, Functional Assessment of Cancer Therapy – Prostate subscale; HbA1c, glycosylated hemoglobin; HDL, high-density lipoprotein; LDL, low-density lipoprotein; MVPA, moderate-to-vigorous physical activity; PSA, prostate-specific antigen.

### Secondary outcomes

Secondary outcomes included quality of life, physical fitness (e.g., upper body strength and lower body function), and blood outcomes and are summarized in **Table 1**.

### Adherence, attrition, and safety

Intervention adherence was defined as achieving at least 150 minutes of objective moderate-to-vigorous physical activity (MVPA) per week. To align with the recent recommendations in physical activity volume in cancer survivors, we also analyzed adherence using a threshold of 90 minutes of MVPA (23). All participants were asked to wear Actigraph GT3X (Pensacola, FL) accelerometers from awaking until bedtime daily for one week prior to each assessment time point, including baseline. Accelerometer data were collected at all assessment time points during the intervention period apart from baseline (pre-intervention). Given the challenges we experienced with obtaining accelerometry data at the 6-month time point, participants were allowed to wear the accelerometer ± 2 weeks from the assessment date (during or just after the intervention period).Weekly wear-time included 500 minutes in at least 4 days (24). Attrition was defined as missing information on both co-primary outcomes at the time of the assessment, similar to previous work (15). Safety was assessed through occurrence of adverse events using the National Cancer Institute Common Terminology Criteria for Adverse Events (CTCAE) version 4.0.

### Sample size and statistical analysis

To calculate our sample, we took a pragmatic approach as we could not ascertain the proportion of eligible participants who would agree to participate, nor we could estimate how many participants would select each preference arm. To maximize study efficiency and yield, we aimed to recruit 30 men in the HOME arm and 40 men in the GROUP arm. Based on these numbers, and using distributional data from our phase II RCT, simulation-based analysis suggested 80% power with ANCOVA for comparing the preference home-based and group arms, respectively, to the equivalent RCT arm for the FACT-F outcome to detect a minimum clinically important difference (MCID) of 4 points (25). Similarly, for the 6MWT, power was just below 80% for the home-based and above 80% for the group-based comparisons (26).

Frequencies and proportions were used to summarize recruitment - overall and by treatment arm - and reasons for refusal to be randomized. Unpaired t-tests or Wilcoxon rank sum tests (for continuous data) and Fisher’s exact tests (for categorical data) were used to compare baseline characteristics between participants in the preference trial and the phase III RCT. Chi-squared tests were used to compare adherence and attrition between the two trials. In the preference study, a random-intercept linear mixed model with an interaction between group and a three-level time variable (baseline, 3 and 6 months) was used to assess the effects of HOME versus GROUP on primary and secondary outcomes; the interaction term at 6 months estimates the difference in change from baseline. To estimate the selection effect (i.e., the effect on study outcomes of being in the preference trial or RCT, regardless of the mode of exercise delivery) and the treatment effect (i.e., effect of each mode of exercise delivery, regardless of randomization) we used a linear regression model with the 6-month measurement as the outcome and the baseline value, study, and arm as predictors. Duration of ADT was not included as a covariate per study protocol due to the small sample size and risk of model overfitting. In this analysis, participants were included only if they had data at baseline and at 6 months, in line with a modified intention-to-treat approach. The analytical approach was modified and published on clinicaltrials.gov prior to analyzing any data. Missing data were not imputed. Selection and treatment effects are illustrated in **Figure 1**. A p-value less than 0.05 was taken to indicate statistical significance. No adjustments for multiple comparisons were made. Estimates are presented with 95% confidence intervals (95%CI). Analyses were done in R 4.2.0.

## Results

### Recruitment

A total of 260 participants were eligible, of whom 54 were recruited into the preference-based trial from October 2017 until April 2020. The recruitment rate was 20.8%. Of the 206 participants who declined to participate, 68% were not interested in an exercise trial, 13.1% provided lack of time as a reason for not participating, whereas 8.7% stated that they preferred to exercise on their own. Additional reasons for declining to participate are shown in **Figure 2**.

### Baseline characteristics of participants in the preference trial and those in the RCT

Analysis of baseline characteristics between the participants in the preference trial and RCT are shown in **Table 2**. Participants in the RCT exhibited higher fatigue, alcohol consumption, total cholesterol, low-density lipoprotein (LDL), and high-density lipoprotein (HDL) compared to participants in the preference trial but were otherwise similar in terms of age, comorbidity, baseline physical fitness and quality of life scores, and other characteristics.

**Table 2:** Baseline characteristics between participants in the preference-based and randomized controlled trials.

Characteristic	Preference-based trial(N=54)	RCT(N=38)	p	Missing %
Age (years), mean (SD)	70.1 (8.6)	70.5 (8.9)	0.86	0.0
Race, n (%)			0.57	0.0
Black	4 (7.4)	3 (7.9)		
East Asian	1 (1.9)	2 (5.3)		
Metis	0 (0)	2 (5.3)		
Oriental	0 (0)	1 (2.6)		
Other	0 (0.0)	1 (2.6)		
South Asian	4 (7.4)	1 (2.6)		
White	45 (83.3)	27 (73.0)		
Marital (Married), n (%)	41 (75.9)	28 (73.7)	0.81	0.0
Education (Completed University/College), n (%)	38 (70.4)	22 (57.9)	0.26	0.0
Work (Retired/semi-retired), n (%)	35 (64.8)	28 (75.7)	0.35	1.1
Distance, median (IQR)	11.7 (8.1-18.8)	12.9 (6.9-30.0)	0.80	8.7
Income (<$60k)^a^	23 (42.6)	18 (48.6)	0.74	1.1
Previously participated in formal exercise program (False), n (%)	41 (75.9)	23 (60.5)	0.16	0.0
Smoking status (Never), n (%)	29 (53.7)	17 (48.6)	0.30	3.3
Drinks per week (1 to 7), n (%)	25 (46.3)	19 (70.4)	**0.014**	12.0
FACT-F, mean (SD)	35.4 (8.3)	31.2 (7.6)	**0.018**	6.5
FACT-G, mean (SD)	80.4 (13.4)	75.6 (13.1)	0.10	8.7
FACT-P subscale, mean (SD)	30.4 (5.9)	29.5 (6.2)	0.46	6.5
Comorbidities				
Previous Myocardial infarction (yes), n (%)	3 (5.6)	1 (2.6)	0.64	0.0
Congestive heart failure (yes), n (%)	0 (0)	0 (0)	1.00	0.0
Peripheral vascular disease (yes), n (%)	0 (0)	0 (0)	1.00	0.0
Angina (yes), n (%)	0 (0)	3 (7.9)	0.067	0.0
Hypertension (yes), n (%)	23 (42.6)	15 (39.5)	0.83	0.0
Atrial fibrillation (yes), n (%)	2 (3.7)	2 (5.3)	1.00	0.0
Hyperlipidemia (yes), n (%)	12 (22.2)	9 (23.7)	1.00	0.0
Other cardiovascular conditions (yes), n (%)	13 (24.1)	8 (21.1)	0.80	0.0
Diabetes (yes), n (%)	6 (11.1)	3 (7.9)	0.73	0.0
Arthritis (yes), n (%)	20 (37.0)	20 (52.6)	0.20	0.0
Other musculoskeletal issues (yes), n (%)	17 (31.5)	10 (26.2)	0.64	0.0
ECOG performance status, n (%)			0.82	6.5
0	14 (25.9)	7 (21.9)		
1	35 (64.8)	23 (71.9)		
2	5 (9.3)	2 (6.2)		
Indication for ADT, n (%)			0.57	12.0
Adjuvant therapy	22 (44.0)	18 (58.1)		
Biochemical failure	15 (30.0)	9 (29.0)		
Metastatic disease	10 (20.0)	3 (9.7)		
Primary treatment	3 (6.0)	1 (3.2)		
Length of ADT, n (%)			0.37	0.0
<3 months	6 (11.1)	7 (18.4)		
>3 months	48 (88.9)	31 (81.6)		
Stage			**0.006**	4.3
T1/T2	28 (51.9)	21 (61.8)		
T3/T4	11 (20.4)	10 (29.4)		
N1	3 (5.6)	0 (0)		
M1	1 (1.9)	3 (8.8)		
Unavailable	11 (20.4)	0 (0)		
Gleason, n (%)			0.95	7.6
2-6	6 (12.0)	5 (14.3)		
7	24 (48.0)	16 (45.7)		
8-10	20 (40.0)	14 (40.0)		
PSA, median (IQR)	0.10 (0.09 – 0.41)	0.10 (0.07 – 0.40)	0.71	20.7
Hemoglobin, mean (SD)	134.4 (13.7)	131.2 (13.2)	0.34	20.7
Total cholesterol (mmol/L), mean (SD)	4.5 (1.2)	5.3 (1.1)	**0.008**	26.1
LDL (mmol/l), mean (SD)	2.6 (1.0)	3.1 (1.1)	**0.046**	26.1
HDL (mmol/L), mean (SD)	1.3 (0.3)	1.6 (0.5)	**0.002**	26.1
Triglycerides (mmol/L), mean (SD)	1.5 (0.7)	1.5 (0.8)	0.96	26.1
Fasting blood glucose (mmol/L), mean (SD)	6.1 (1.3)	5.8 (1.2)	0.43	30.4
Testosterone (nmol/L), mean (SD)	1.0 (3.0)	0.8 (2.0)	0.75	26.1
HbA1c, (%), mean (SD)	5.9 (1.0)	5.8 (0.5)	0.57	31.5

a Lowest option for annual income.

ADT, androgen deprivation therapy; FACT-F, Functional Assessment of Cancer Therapy – Fatigue; FACT-G, Functional Assessment of Cancer Therapy – General; FACT-P subscale, Functional Assessment of Cancer Therapy – Prostate subscale; ECOG, Eastern Cooperative Oncology Group; HbA1C, hemoglobin A1c; HDL, high-density lipoprotein; IQR, interquartile range; km, kilometers; LDL, low-density lipoprotein; PSA, prostate-specific antigen; SD, standard deviation. Bold values represent statistically significant results.

The characteristics of GROUP and HOME participants of the preference trial are shown in (**Table 3**). GROUP participants were living in closer proximity to the study centers compared to HOME participants (median difference: -6km, 95%CI= -10 to 0, *p*=0.042).

**Table 3:** Characteristics of participants in the preference trial at baseline.

Characteristic	Overall(N=54)	Group(n=17)	Home(n=37)	p	Missing %
Age (years), mean (SD)	70.2 (8.6)	73.2 (7.2)	68.7 (9.0)	0.074	0.0
Race n (%)				0.27	0.0
Black	4 (7.4)	3 (17.6)	1 (2.7)		
East Asian	1 (1.9)	0 (0)	1 (2.7)		
South Asian	4 (7.4)	1 (5.9)	3 (8.1)		
White	45 (83.3)	13 (76.5)	32 (86.5)		
Married, n (%)	41 (75.9)	10 (58.8)	31 (83.8)	0.084	0.0
Education (Completed University/College), n (%)	38 (70.4)	12 (70.6)	26 (70.3)	1.00	0.0
Work (Retired/semi-retired), n (%)	35 (64.8)	12 (70.6)	23 (62.2)	0.76	0.0
Distance (km), median (IQR)	11.7 (8.1-18.8)	8.8 (4.9-15.6)	12.1 (10.4-22.6)	**0.042**	14.8
Income (<$60k)^a^	23 (42.6)	10 (58.8)	13 (35.1)	0.18	0.0
Never previously participated in formal exercise program, n (%)	41 (75.9)	11 (64.7)	30 (81.1)	0.30	0.0
Never smoked, n (%)	29 (53.7)	11 (64.7)	18 (48.6)	0.51	0.0
Drinks per week (1 to 7), n (%)	25 (46.3)	8 (47.1)	17 (45.9)	0.70	0.0
FACT-F, mean (SD)	35.4 (8.3)	36.0 (7.4)	35.1 (8.6)	0.74	11.1
FACT-G, mean (SD)	80.4 (13.4)	78.8 (11.0)	81.1 (14.4)	0.59	13.0
FACT-P subscale, mean (SD)	30.4 (5.9)	28.8 (5.6)	31.2 (6.0)	0.20	9.3
Comorbidities					
Previous Myocardial infarction, n (%)	3 (5.6)	2 (11.8)	1 (2.7)	0.23	0.0
Congestive heart failure, n (%)	0 (0)	0 (0)	0 (0)	1.00	0.0
Peripheral vascular disease, n (%)	0 (0)	0 (0)	0 (0)	1.00	0.0
Angina, n (%)	0 (0)	0 (0)	0 (0)	1.00	0.0
Hypertension, n (%)	23 (42.6)	7 (41.2)	16 (43.2)	1.00	0.0
Atrial fibrillation, n (%)	2 (3.7)	1 (5.9)	1 (2.7)	0.53	0.0
Hyperlipidemia, n (%)	12 (22.2)	4 (23.5)	8 (21.6)	1.00	0.0
Other cardiovascular conditions, n (%)	13 (24.1)	4 (23.5)	9 (24.3)	1.00	0.0
Diabetes, n (%)	6 (11.1)	3 (17.6)	3 (8.1)	0.36	0.0
Arthritis, n (%)	20 (37.0)	7 (41.2)	13 (35.1)	0.76	0.0
Other musculoskeletal issues, n (%)	17 (31.5)	7 (41.2)	10 (27.0)	0.35	0.0
ECOG performance status, n (%)				0.53	0.0
0	14 (25.9)	3 (17.6)	11 (29.7)		
1	35 (64.8)	13 (76.5)	22 (59.5)		
2	5 (9.3)	1 (5.9)	4 (10.8)		
Indication for ADT, n (%)				0.22	7.4
Adjuvant therapy	22 (44.0)	6 (37.5)	16 (47.1)		
Biochemical failure	15 (30.0)	3 (18.8)	12 (35.3)		
Metastatic disease	10 (20.0)	5 (31.2)	5 (14.7)		
Primary treatment	3 (6.0)	2 (12.5)	1 (2.9)		
Length of ADT, n (%)				1.00	0.0
<3 months	6 (11.1)	2 (11.8)	4 (10.8)		
>3 months	48 (88.9)	15 (88.2)	33 (89.2)		
Stage				0.053	0.0
T1/T2	28 (51.9)	6 (35.3)	22 (59.5)		
T3/T4	11 (20.4)	3 (17.6)	8 (21.6)		
N1	3 (5.6)	3 (17.6)	0 (0)		
M1	1 (1.9)	0 (0)	1 (2.7)		
Unavailable	11 (20.4)	5 (29.4)	6 (16.2)		
Gleason, n (%)				0.58	7.4
2-6	6 (12.0)	1 (6.2)	5 (14.7)		
7	24 (48.0)	7 (43.8)	17 (50.0)		
8-10	20 (40.0)	8 (50.0)	12 (35.3)		
PSA, median (IQR)	0.10 (0.09 - 0.41)	0.10 (0.08 – 0.27)	0.10 (0.10 – 0.47)	0.89	14.8
Hemoglobin, mean (SD)	134.5 (13.7)	131.9 (13.8)	135.7 (13.7)	0.37	9.3
Total cholesterol (mmol/L), mean (SD)	4.5 (1.2)	4.5 (1.4)	4.5 (1.1)	0.92	14.8
LDL (mmol/l), mean (SD)	2.6 (1.0)	2.7 (1.0)	2.5 (1.0)	0.51	14.8
HDL (mmol/L), mean (SD)	1.3 (0.3)	1.3 (0.3)	1.3 (0.2)	0.92	14.8
Triglycerides (mmol/L), mean (SD)	1.5 (0.7)	1.8 (0.8)	1.4 (0.6)	0.10	14.8
Fasting blood glucose (mmol/L), mean (SD)	6.1 (1.3)	6.1 (0.8)	6.1 (1.5)	0.98	16.7
Testosterone (nmol/L), mean (SD)	1.0 (3.0)	0.4 (0.2)	1.2 (3.6)	0.42	20.4
HbA1c, (%), mean (SD)	5.9 (1.0)	5.6 (0.6)	6.1 (1.1)	0.15	20.4

a Lowest option for annual income.

ADT, androgen deprivation therapy; ECOG, Eastern Cooperative Oncology Group; FACT-F, Functional Assessment of Cancer Therapy – Fatigue; FACT-G, Functional Assessment of Cancer Therapy – General; FACT-P subscale, Functional Assessment of Cancer Therapy – Prostate subscale; HbA1C, hemoglobin A1c; HDL, high-density lipoprotein; IQR, interquartile range; km, kilometers; LDL, low-density lipoprotein; PSA, prostate-specific antigen; SD, standard deviation. Only one participant was lost to follow up during the study and thus baseline comparisons with those retained were not performed due to the small sample. Bold values represent statistically significant results.

### Effects of HOME and GROUP on primary and secondary outcomes in preference participants

Baseline and 6-month mean values of primary and secondary outcomes, and model-based estimates of differences between HOME and GROUP in the mean changes from baseline to 6 months are presented in **Table 4**. No significant differences between HOME and GROUP in the change from baseline were found in any of the outcomes.

**Table 4:** Differences in changes from baseline to 6 months in primary and secondary outcomes (preference-based participants only).

Outcomes	Arm	n(BL/6m)	Baseline	6 Months	Model-based difference (HOME-GROUP) in mean change(95%CIs)	*P*
*Primary*						
FACT-F	Home	35/28	35.1 (8.6)	40.8 (8.8)	4.4 (-1.7 to 10.6)	0.16
	Group	13/11	36 (7.4)	36.9 (7.5)		
6MWT (m)	Home	37/28	520.6 (110.1)	597.6 (105.3)	12.8 (-68.1 to 42.6)	0.65
	Group	17/10	548.1 (102.3)	615.4 (71.4)		
*Secondary*						
*Physical fitness and body composition outcomes*						
Chair Stands (s)	Home	37/28	12.8 (5.7)	9.0 (2.3)	0.4 (-1.3 to 2.1)	0.64
	Group	17/11	11.6 (4.3)	8.4 (3.2)		
Grip Strength (kg)	Home	37/28	40.9 (11.1)	43.8 (9.7)	-1.5 (-4.7 to 1.7)	0.37
	Group	17/11	36.8 (8.5)	38.0 (9.3)		
Body Mass Index (kg/m2)	Home	37/28	29.0 (4)	28.8 (4)	-0.3 (-0.9 to 0.3)	0.37
	Group	17/11	27.6 (3.6)	28.3 (2.9)		
Waist Circumference (cm)	Home	37/28	106.1 (11)	103.9 (9.6)	-0.8 (-3.5 to 1.8)	0.54
	Group	17/11	100.8 (9.6)	102.7 (8.2)		
Hip Circumference (cm)	Home	37/28	105.8 (6.8)	106.3 (7.7)	-1.1 (-3.4 to 1.2)	0.35
	Group	16/11	100.8 (5.5)	101.7 (6.2)		
Waist : Hip ratio	Home	37/28	1.0 (0.1)	1.0 (0.1)	0 (0 to 0)	0.69
	Group	16/11	1.0 (0.1)	1.0 (0.1)		
Fat Mass (kg)	Home	35/28	25.2 (9.5)	25.3 (10)	0.4 (-1.4 to 2.2)	0.66
	Group	17/11	22.6 (10.9)	22.4 (5.7)		
Fat-free Mass (kg)	Home	35/28	61.8 (6.9)	61.9 (7.5)	-0.1 (-1.7 to 1.5)	0.87
	Group	17/11	55.8 (12.9)	59.7 (5.9)		
Body Fat (%)	Home	35/28	28.4 (5.9)	28.3 (6.4)	0.7 (-0.9 to 2.2)	0.41
	Group	17/11	25.0 (4.8)	26.9 (4.3)		
*Patient-reported outcomes*						
FACT-G	Home	33/28	81.1 (14.4)	83.4 (13.6)	4 (-2.1 to 10.1)	0.20
	Group	14/11	78.8 (11.0)	77.7 (8.3)		
FACT-P subscale	Home	34/28	31.2 (6.0)	30.8 (6.8)	0.2 (-3.2 to 3.6)	0.91
	Group	15/11	28.8 (5.6)	27.9 (7.1)		
*Blood markers*						
Log_10_ (PSA) (µg/L)	Home	32/25	-0.8 (0.7)	-0.8 (1)	0.1 (-0.3 to 0.5)	0.75
	Group	14/10	-0.6 (1.1)	-1 (0.7)		
Hemoglobin (g/L)	Home	33/27	135.7 (13.7)	139.4 (13.7)	2.5 (-3.4 to 8.4)	0.41
	Group	16/10	131.9 (13.8)	134.9 (13.2)		
Total Cholesterol (mmol/L)	Home	31/27	4.5 (1.1)	4.7 (1.3)	0.1 (-0.5 to 0.6)	0.74
	Group	15/10	4.5 (1.4)	4.4 (1.5)		
LDL (mmol/L)	Home	31/27	2.5 (1)	2.8 (1.1)	0.1 (-0.3 to 0.6)	0.62
	Group	15/10	2.7 (1)	2.6 (1.2)		
HDL (mmol/L)	Home	31/27	1.3 (0.3)	1.3 (0.4)	-0.1 (-0.4 to 0.1)	0.35
	Group	15/10	1.3 (0.3)	1.4 (0.7)		
Triglycerides (mmol/L)	Home	31/27	1.4 (0.6)	1.4 (0.7)	0.2 (-0.3 to 0.6)	0.50
	Group	15/10	1.8 (0.8)	1.7 (0.7)		
Blood glucose (mmol/L)	Home	31/27	6.1 (1.5)	5.8 (1)	-0.2 (-0.7 to 0.4)	0.55
	Group	14/10	6.1 (0.8)	6.1 (0.9)		
Hemoglobin A1c (%)	Home	30/26	6.1 (1.1)	5.8 (0.7)	-0.1 (-0.3 to 0.1)	0.37
	Group	13/11	5.6 (0.6)	5.6 (2.3)		

6MWT, 6-minute walk test; BL, baseline; BMI, body mass index; FACT-F, Functional Assessment of Cancer Therapy – Fatigue; FACT-G, Functional Assessment of Cancer Therapy – General; FACT-P subscale, Functional Assessment of Cancer Therapy – Prostate subscale; HDL, high-density lipoprotein; LDL, low-density lipoprotein; PSA, prostate-specific antigen; WC, waist circumference.

### Adherence and attrition between the preference trial and the RCT

Adherence was similar between participants in the preference trial and the RCT regardless of the MVPA threshold used (i.e., 150mins (p= 0.81) or 90mins (p=0.68) MVPA) as shown in **Table 5**. Attrition was higher in the RCT compared to the preference-based trial at 6 months (50.0% vs. 27.8%, p=0.04). However, no significant differences were found in attrition by study arm between the two studies, likely due to the small sample. Specifically, retention for GROUP at 6 months was 64.7% and 50.0% in the preference trial and RCT, respectively (p=0.51). Retention for HOME at 6 months was 78.4% in the preference trial and 50.0% in the RCT (p=0.06).

**Table 5:** Adherence between preference and RCT participants.

Baseline	GROUP : Preference	GROUP : RCT	HOME : Preference	HOME : RCT	P
n	16	19	34	18	
≥150mins MVPA, n (%)	5 (31.2)	5 (35.7)	10 (29.4)	1 (6.2)	0.22
≥ 90mins MVPA, n (%)	9 (56.2)	6 (42.9)	15 (44.1)	3 (18.8)	0.17
*6 months*					
≥150mins MVPA, n (%)	5 (45.5)	3 (30.0)	7 (29.2)	2 (33.3)	0.81
≥ 90mins MVPA, n (%)	8 (72.7)	5 (50.0)	13 (54.2)	3 (50.0)	0.68

Mins, minutes; MVPA, moderate-to-vigorous physical activity; RCT, randomized controlled trial.

In the preference-based trial at 6 months, retention was 72.7% and adherence (i.e., at least 150 minutes of MVPA per week) was 37.1%; 62.9% of preference participants adhered to the updated threshold of physical activity guidelines (i.e., 90 minutes of MVPA per week). Comparisons between GROUP and HOME did not reveal differences in MVPA at baseline (p= 0.70) or 6 months (p=0.33). Attrition at 6 months was not different between GROUP and HOME in the preference-based trial (p=0.51). Retention was numerically higher in HOME (78.4%) than GROUP (64.7%) at 6 months but did not reach statistical significance (p=0.32).

### Safety in the preference trial

In total, 7 adverse events (4 Grade 2 and 3 Grade 3) were reported during the intervention (see **Supplementary Material**). Three out of seven adverse events (one Grade 2, two Grade 3) were judged by the investigators to be possibly related to the intervention.

### Selection and treatment overall effects in combined preference trial and RCT

**Figure 3:**
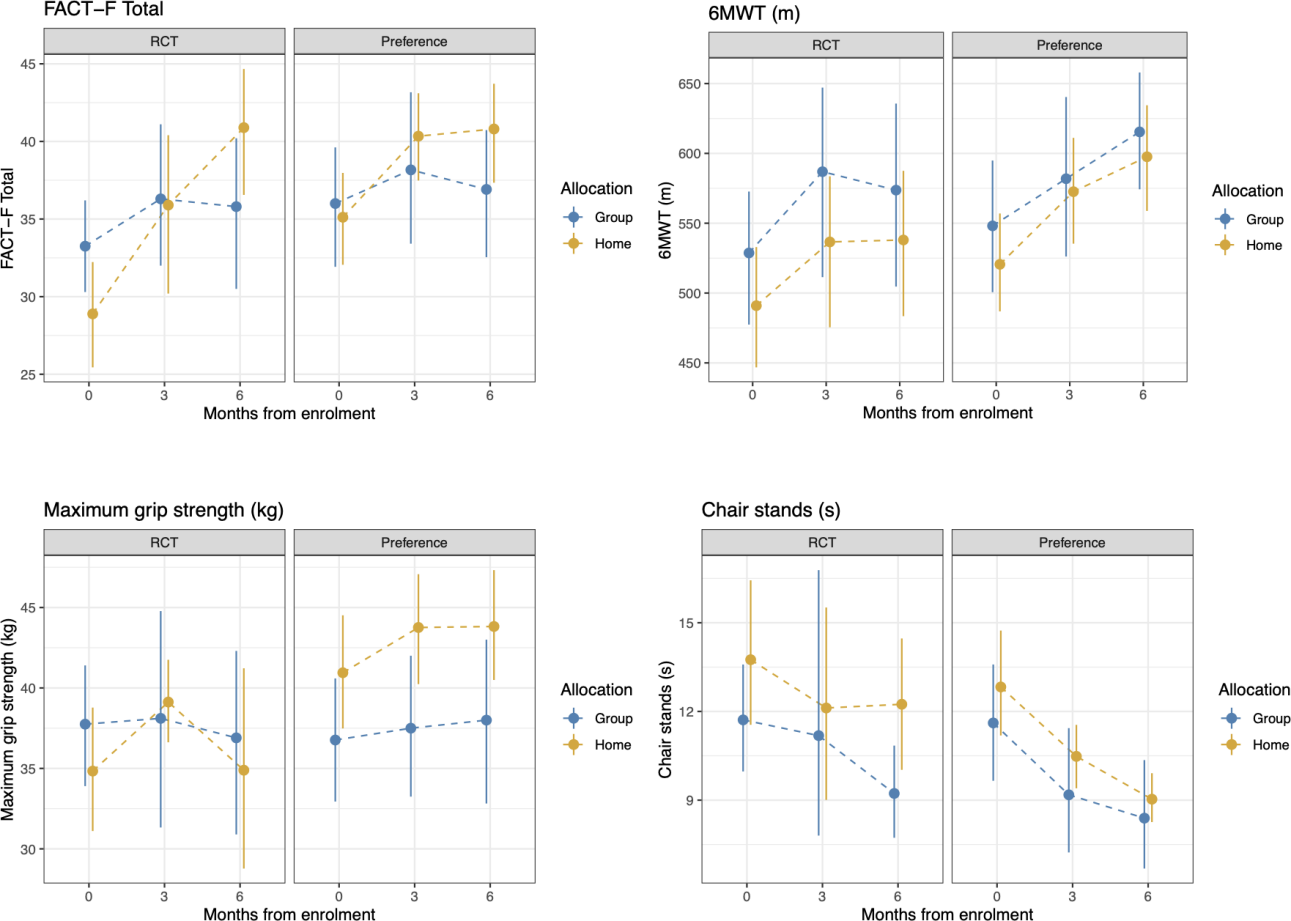
Changes in FACT-F, 6MWT, grip strength, and chair stands from baseline to 6 months between HOME and GROUP participants in the RCT and the preference trial. Higher scores represent less fatigue, greater walk distance, greater grip strength, and longer time to complete the chair stands test.

**Figure 4:**
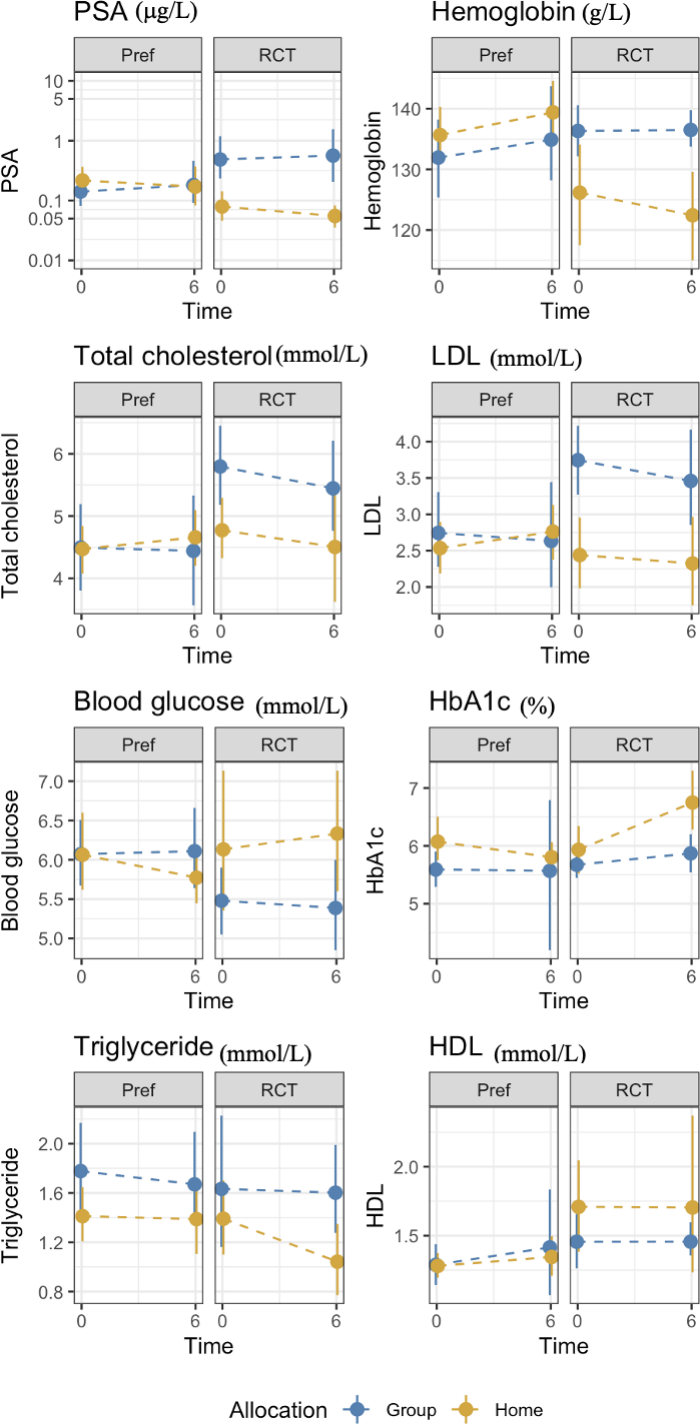
Changes in blood markers between from baseline to 6 months HOME and GROUP participants in the preference trial and the RCT.

**Table 6** shows the selection effect. Preference trial participants covered a greater distance in the 6MWT (mean difference: 28.7 meters, 95% CI= -15.6 to 72.9) than their counterparts in the RCT at 6 months; however, this difference did not reach statistical significance (p=0.20). Similarly, no significant differences were observed for fatigue between the two trials (mean difference: -2.4, 95%CI= -6.6 to 1.8 *p*=0.25). Preference trial participants performed chair stands 1.2 seconds faster compared to RCT participants at 6 months, a difference of marginal statistical significance (p=0.06). Compared to RCT participants, preference trial participants exhibited a significant increase in hip circumference at 6 months (mean difference 2.9 cm, 95%CI= 0.4 to 5.5, p=0.02). A significant reduction in serum hemoglobin was found for preference participants compared to their RCT counterparts (mean difference: -7.1 g/L, 95%CI= -13.0 to -1.3, p=0.01). No significant differences were observed for any other outcomes. **Figures 3**, **4** show changes in fatigue, physical fitness, and blood outcomes between preference and RCT participants by study arm.

**Table 6:** Overall selection and intervention effects between trials and study arms for outcomes without potential differential effect.

Outcome	Selection effect		Intervention effect (Preference trial and RCT)	
	**Preference trial vs. RCT^a^** **Mean (95%CIs)**	***P***	**HOME vs. GROUP^b^** Mean (95%CIs)	***P***
*Primary*				
FACT-F, units	-2.4 (-6.6 to 1.8)	0.25	5.2 (1.3 to 9.2)	**0.01**
6MWT (m)	28.7 (-15.6 to 72.9)	0.20	-3.8 (48.8 to 39.3)	0.86
*Secondary*				
*Physical fitness and body composition outcomes*				
Grip strength (kg)	0.5 (-3.1 to 4.2)	0.78	2.5 (-0.9 to 6.0)	0.14
Chair stands (s)	-1.2 (-2.5 to 0)	0.06	0.8 (-0.4 to 2.0)	0.21
BMI (kg/m2)	0.3 (-0.30 to 0.9)	0.32	-0.1 (-0.7 to 0.5)	0.76
Waist circumference (cm)	-0.2 (-2.6 to 2.1)	0.86	-0.3 (-2.6 to 2.1)	0.83
Hip circumference (cm)	2.9 (0.4 to 5.5)	**0.02**	-0.4 (-2.8 to 2.0)	0.76
Waist:hip ratio	0.0 (0 to 0)	0.29	0.0 (0 to 0)	0.50
Fat mass (kg)	-0.2 (-1.7 to 1.2)	0.75	-0.7 (-2.1 to 0.8)	0.36
Fat-free mass (kg)	0.1 (-1.1 to 1.4)	0.82	-0.1 (-1.3 to 1.2)	0.91
Body fat (%)	-0.2 (-1.4 to 1.1)	0.78	-0.6 (-1.8 to 0.6)	0.33
*Patient-reported outcomes*				
FACT-G	3.8 (-4.3 to 12.0)	0.35	2.4 (-5.6 to 10.3)	0.55
FACT-P subscale	-0.9 (-3.8 to 2.0)	0.54	0.7 (-2.2 to 3.5)	0.63
*Blood markers*				
PSA (µg/L)	-0.1 (-0.4 to 0.3)	0.75	-0.1 (-0.4 to 0.3)	0.71
Hemoglobin (g/L)	-7.1 (-13.0 to -1.3)	**0.01**	1.5 (-3.6 to 6.5)	0.56
Total cholesterol (mmol/L)	0.1 (-0.5 to 0.6)	0.82	0 (-0.5 to 0.5)	0.98
LDL (mmol/L)	0 (-0.4 to 0.4)	0.91	0 (-0.3 to 0.3)	0.96
HDL (mmol/L)	-0.1 (-0.4 to 0.3)	0.60	-0.1 (-0.3 to 0.2)	0.65
Triglycerides (mmol/L)	-0.1 (-0.4 to 0.3)	0.65	-0.1 (-0.4 to 0.2)	0.52
Blood glucose (mmol/L)	-0.1 (-0.6 to 0.4)	0.73	-0.1 (-0.6 to 0.3)	0.58
HbA1c (%)	0.3 (0.0 to 0.6)	0.05	0 (-0.2 to 0.3)	0.81

6MWT, 6-minute walk test; BMI, body mass index; FACT-F, Functional Assessment of Cancer Therapy – Fatigue; FACT-G, Functional Assessment of Cancer Therapy – General; FACT-P subscale, Functional Assessment of Cancer Therapy – Prostate subscale; HbA1c, hemoglobin A1c; HDL, high-density lipoprotein; LDL, low-density lipoprotein; PSA, prostate-specific antigen; RCT, randomized controlled trial; WC, waist circumference.

The number of participants for each outcome by study arm is included in **Supplementary Material**. Bold values represent statistically significant results.

The analysis of the treatment effect (GROUP or HOME regardless of trial design) demonstrated that the HOME arms reported lower fatigue at 6 months than the GROUP participants (mean difference: 5.2 points, 95%CI= 1.3 to 9.2, p= 0.01) with no significant differences in other outcomes (**Table 6**).

## Discussion

We compared multiple characteristics of patients willing to be enrolled in a preference trial, to those in a randomized study, and compared the effects of a preference-based exercise modality on clinically relevant outcomes in PC survivors on ADT against a RCT of the same exercise interventions. By doing so, we provide insights into advantages and drawbacks of preference trials compared to RCTs.

Participants approached to participate in the preference trial were those who declined to participate in an RCT of the same interventions. As we offered enrolment in the RCT first then the preference trial, we cannot directly determine whether recruitment is better in a preference trial. What is noteworthy is that the option of preference selection led 20.8% of participants to participate in the preference trial after declining participation in the RCT where recruitment was poor (6.7%). This suggests that allowing participants to select their arm of preference fosters recruitment in a group of patients that refused to participate in a RCT of the same interventions. Evidence from two systematic reviews (14, 15) and a meta-analysis (14) suggests that treatment preferences predispose many participants to refuse randomization, thereby undermining recruitment in RCTs. Whether the element of preference can counteract exercise-related barriers in PC survivors on ADT [e.g., dramatic declines in QOL (27, 28), fatigue (29), reduced motivation (29)] requires further exploration.

Assessment of baseline characteristics revealed that participants who were randomized exhibited higher fatigue, higher alcohol consumption, and a less favorable lipid profile than their counterparts in the preference trial. No differences were observed in other sociodemographic or clinical characteristics at baseline. Such differences in fatigue and other lifestyle or metabolic variables (e.g., smoking and lipid profile) further highlight the need for these patients to engage in exercise to improve their energy levels and metabolic health, given that those with poor physical fitness are likely to benefit the most from exercise (30). Therefore, providing these participants with the option to select their preferred exercise type and/or mode may optimize adherence, leading to the adoption of an exercise routine through which patients can experience several health benefits. Previous work in mixed clinical populations have also found comparable clinical and sociodemographic characteristics among randomized and preference groups (14, 15). Thus, given the lack of differences between preference and RCT participants in most characteristics at baseline, and the impact of participant preferences on recruitment, preference trials may improve external validity (15).

Participants in the preference-based trial exhibited lower attrition at 6 months compared with RCT participants. Our findings are novel in oncology and support the theory that participant preferences may foster motivation but warrant further research in men with PC on ADT, as well as patients with other types of cancer. Meta-analytic evidence of 20 studies in mixed clinical populations including cancer found that loss to follow up was significantly greater in RCTs than preference cohorts (14). Nonetheless, others did not observe an influence of participant preferences on attrition (15). An attempt to compare our findings of attrition to those of previous work (14, 15) would be misleading given the population heterogeneity. Cancer is associated with profound psychological and physical changes that differ from other clinical populations. Existing evidence does not capture important and common consequences of a cancer diagnosis or treatment (e.g., emotional, physical, lifestyle changes) that are important to better understand whether participant preferences impact attrition in exercise trials. Therefore, further research is warranted in homogenous populations in terms of cancer type and treatment given the lack of preference trials in the area of exercise in oncology and the theoretical rationale for offering to participants the option to select their preferred mode of exercise as a strategy to foster motivation and potentially intervention adherence. Retention by study arms between the two studies revealed potentially important differences that did not reach statistical significance, likely due to the small sample. For example, 64.7% of participants in the preference trial who underwent GROUP remained in the study at 6 months, whereas retention of GROUP in the RCT at 6 months was 50.0%. Similar results were found for HOME between the two studies. When GROUP and HOME were compared in terms of retention in the preference trial at 6 months, HOME was found to be superior. Although motivational readiness was not examined, these findings suggest that participants with a preference are more likely to remain in an exercise study than those who are randomized. A second conclusion which requires further research is that HOME may optimize retention compared to GROUP, which may be relevant to program development for research and clinical purposes.

Additionally, compared to the RCT, no significant differences were found in intervention adherence. Our findings are not in line with previous research that observed greater adherence in patients with knee osteoarthritis who were assigned to their preferred choice of program compared with those who did not (18). However, our population has distinct characteristics and therefore comparisons with other clinical populations may not be appropriate for adherence or attrition. Adherence in patients with cancer may be influenced by numerous factors, such as lack of time due to medical appointments, reduced energy levels due to cancer treatment, particularly systemic therapy, tumor- and treatment-related complications, in addition to changes in mood and overall QOL. Regardless of randomization, participants in both studies might have participated in either the preference trial or RCT because of the well-established health benefits of exercise. However, the lack of a significant difference in adherence between participants in the preference trial and RCT may be attributed to disease- and treatment-related factors. For example, it is possible that fatigue or other symptoms due to ADT hindered the ability of participants with a preference to exercise, despite potential higher motivation readiness.

Our results demonstrated that selection of exercise mode did not improve most physical fitness, patient-reported, or blood outcomes compared to randomization. Participants in the preference trial exhibited a non-significant increase in the 6MWT distance (+28.7 meters, *p*=0.20) compared to their RCT counterparts, a difference that is in line with the MCID for the 6MWT (31). The reduced time in chair stands in favor of preference participants at 6 months did not reach statistical or clinical significance. A significant increase in hip circumference was found for preference participants compared with RCT participants at 6 months. However, no differences were noted in other body composition or anthropometric outcomes, making this outcome difficult to interpret. Additionally, we found that hemoglobin was significantly decreased in preference participants compared with RCT participants at 6 months, but this finding may be spurious due to lack of biological plausibility and small sample size. Meta-analytic data of 20 trials in mixed clinical populations (14) demonstrated comparable treatment effects on outcomes between preference and randomized groups, concluding that participant preferences do not compromise intervention efficacy. Our findings are in line with those found by Wasmann and colleagues (14), suggesting that participants’ preferences do not lead to greater intervention effects compared to a RCT of the same interventions. However, given our modest sample, numerical differences in adherence in favor of preference-based interventions, and the lack of assessing motivational readiness of participants, particularly those who declined participation in both studies, further studies in oncology are warranted.

Regarding the effects of HOME and GROUP from baseline to 6 months irrespective of randomization, we found that participants who underwent GROUP exercise reported worse fatigue relative to HOME participants. A meta-analysis of 31 RCTs in oncology suggested that supervised exercise interventions are more effective in reducing fatigue compared to unsupervised exercise programs (32). The authors suggested the superiority of supervised exercise on fatigue compared to HOME may be explained by positive feedback on progress by qualified exercise professionals during exercise sessions, access to proper exercise equipment, and better adherence (32). In our study, GROUP and HOME participants did not have access to traditional strength training apparatus (e.g., chest press machine, seated row machine, leg press machine), but performed all resistance exercises using free weights and elastic bands. Additionally, our HOME participants were further supported by a health coach and had a fitbit which provided feedback. This additional support in HOME participants likely moderated the differences in fatigue and other outcomes compared with GROUP.

To our knowledge, this is the first study in PC survivors on ADT that attempted to elucidate whether strong preference to an exercise arm impacts intervention efficacy compared with random allocation. An additional strength is the novel comparison of multiple outcomes (e.g., intervention adherence, retention, quality of life, and physical fitness outcomes) between participants in the preference trial and their RCT counterparts. Important limitations of this study include a modest sample size (which limits our ability to make strong statements about the similarity of adherence, retention and outcomes between the two study types) and the high attrition, particularly in the RCT. Despite that most baseline sociodemographic and clinical characteristics were comparable between participants of both studies, RCT participants reported worse fatigue compared to preference participants at baseline. We are unable to determine whether fatigue hindered the ability of some RCT participants to adhere to the intervention or whether some RCT participants who adhered to the intervention experienced greater improvement in fatigue as a result of exercise. It is likely therefore that some of the exercise-related benefits were underestimated in the preference trial. Nonetheless, no significant differences in fatigue were found at 6 months between participants in the preference trial and those in the RCT. Lastly, selection bias in an inherent limitation of our study by design.

## Conclusion

An exercise preference-based trial results in recruitment of 1 in 4 men who declined participation in a RCT of similar interventions in the area of exercise and oncology. Intervention adherence is not influenced by exercise preferences. However, a preference-based trial may lead to lower attrition compared with a RCT of the same interventions. Additionally, selection of an exercise arm may lead to comparable or improved patient-reported, physical fitness, and blood outcomes compared with randomization. HOME exercise may be more effective than GROUP for mitigating fatigue in men with PC. Whether our findings are generalizable to other cohorts should be further investigated.

## Data availability statement

The raw data supporting the conclusions of this article will be made available by the authors, without undue reservation upon approval of the Research Ethics Board.

## Ethics statement

The studies involving human participants were reviewed and approved by Research Ethics Boards at the University Health Network, Tom Baker Cancer Centre, Scarborough, and Rouge Hospital. The patients/participants provided their written informed consent to participate in this study.

## Author contributions

SA, DS, PR, CS, AM, JC, SS, and NC-R: conceptualization. SD, NC-R, and JC: data collection. GT, SA, and EP: data analysis and interpretation. EP: drafting original version. SA: supervision. All authors read, edited, and approved the final version of this manuscript.
