# Supplementary material for: Preference-based versus randomized controlled trial in prostate cancer survivors: Comparison of recruitment, adherence, attrition, and clinical outcomes
Source: Front Oncol. 2022 Dec 12;12:1033229. doi: 10.3389/fonc.2022.1033229 (PMC9791189; doi:10.3389/fonc.2022.1033229)

Supplementary Material

**Supplementary Table 1: Adverse events of participants in the preference trial**

| **Description** | **Site** | **Time point** | **Did it occur during exercise?** | **Classification**  **CTCAE 4.0** | **Determination of cause** |
| --- | --- | --- | --- | --- | --- |
| Increase in vertigo symptoms (n=1) | Calgary | 0-3 months | No | 3 (severe vertigo leading to bedrest for several days) | Possibly related to the intervention (Pre-existing vertigo) |
| Difficulty with dizziness and mobility (n=1) | Calgary | 0-3 months | No | 2 | Not related to the intervention |
| Lost motivation due to 2 falls (n=1) | Calgary | 0-3 months | No | 2 | Not related to the intervention (fell on ice x 2) |
| Referred to cardiology due to arrhythmia (n=1) | UHN | 0-3 months | No | 3  (Symptomatic, participant went to ER where he was instructed to see a cardiologist asap. In the ER, he had an ECG + echocardiogram) | Possibly related to the intervention (participant reported elevated heart rate + atrial fibrillation despite being on beta blockers. Cardiologist changed patient’s meds and was considering ablation procedure. |
| Fainting episode (n=1) | UHN | 3 months | No | 3  (Investigations required, exercise temporarily on hold, cardiology consult) | Not related to the intervention  (Participant experienced a fainting episode in a religious venue. |
| Withdrew due to back pain (n=1) | UHN | 3 months | No | 2 | Not related to the intervention  (Participant had a fractured rib during a chiropractic assessment and withdrew due to pain) |
| Re-injured hernia (n=1) | Calgary | 6 months | No | 2 | Possibly related to the intervention |

Abbreviations: CTCAE=Common Toxicity Criteria for Adverse Events; ECG= electrocardiogram; ER= Emergency Room; UHN=University Health Network

**Supplementary Table 2. Number of participants included in the analyses of the selection and treatment effect**

|  | Preference | | RCT | |
| --- | --- | --- | --- | --- |
| Outcome | Home (n) | Group (n) | Home (n) | Group (n) |
| Primary |  |  |  |  |
| FACT-F Total | 27 | 10 | 9 | 10 |
| 6MWT (m) | 28 | 10 | 8 | 10 |
| Secondary |  |  |  |  |
| *Physical fitness and body composition outcomes* | | | | |
| Maximum grip strength (kg) | 28 | 11 | 9 | 10 |
| Chair stands (s) | 28 | 11 | 8 | 10 |
| BMI (kg/m^2) | 28 | 11 | 9 | 10 |
| Waist circumference (cm) | 28 | 11 | 9 | 10 |
| Hip circumference (cm) | 28 | 11 | 9 | 10 |
| Waist:hip ratio | 28 | 11 | 9 | 10 |
| Fat mass (kg) | 28 | 11 | 8 | 10 |
| Fat free mass (kg) | 28 | 11 | 8 | 10 |
| Body fat (%) | 28 | 11 | 8 | 10 |
| *Patient-reported outcomes* | | | | |
| FACT-G Total | 26 | 10 | 9 | 10 |
| FACT-P Subscale | 27 | 11 | 9 | 10 |
| *Blood markers* | | | | |
| PSA (µg/L) | 22 | 9 | 7 | 7 |
| Hemoglobin (g/L) | 25 | 10 | 5 | 4 |
| Total cholesterol (mmol/L) | 22 | 10 | 5 | 5 |
| LDL (mmol/L) | 22 | 10 | 5 | 5 |
| HDL (mmol/L) | 22 | 10 | 5 | 5 |
| Triglycerides (mmol/L) | 22 | 10 | 5 | 5 |
| Blood glucose (mmol/L) | 22 | 10 | 5 | 4 |
| HbA1c (%) | 22 | 9 | 4 | 4 |

6MWT= 6-minute walk test; BMI= body mass index; FACT-F= Functional Assessment of Cancer Therapy – Fatigue; FACT-G= Functional Assessment of Cancer Therapy – General; FACT-P subscale= Functional Assessment of Cancer Therapy – Prostate subscale; HbA1c= hemoglobin A1c; HDL= high-density lipoprotein; LDL= low-density lipoprotein; PSA= prostate-specific antigen; RCT= randomized controlled trial; WC= waist circumference


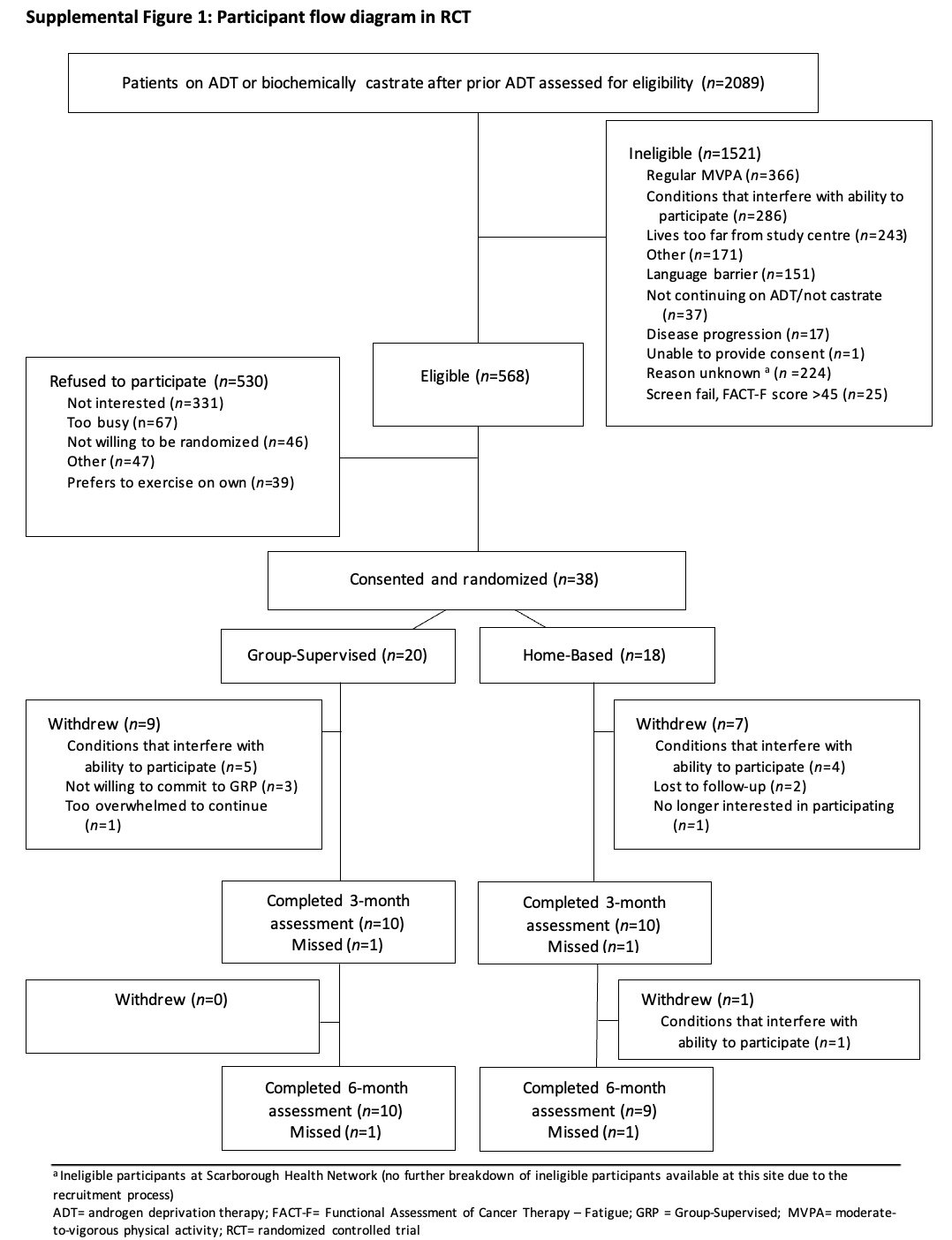

Supplement: Supplementary file 1 [file DataSheet_1.docx]
